# Social Media Use and the Quality of Family Relationships: A Six-Wave Longitudinal Study of Within-Family Effects from Age 10 to 20

**DOI:** 10.1007/s10964-026-02362-5

**Published:** 2026-06-04

**Authors:** Habib Niyaraq Nobakht, Lars Wichstrøm, Silje Steinsbekk

**Affiliations:** 1 Department of Psychology, Norwegian University of Science and Technology (NTNU), Trondheim, Norway; 2 St Olavs Hospital, The Department of Mental Health – Acute, Elderly, Child and Adolescent Services, Department of Child and Adolescent Psychiatry, Trondheim, Norway

**Keywords:** Social Media Use, Family Relationships, Family Climate, Family Functioning, Within-Family Analysis

## Abstract

Adolescents and adults spend several hours a day on social media, raising concerns that extensive social media use may negatively affect family life. However, it remains unclear whether social media use is associated with changes in family relationship quality, as prior research has not separated stable between-family differences from within-family prospective effects and has often relied on single-informant data. These limitations risk inflating associations through shared methods variance and invite misinterpretation of cross-sectional links as developmental processes. Data were drawn from two birth cohorts from Trondheim, Norway, followed across six biennial waves from ages 10 to 20 (*n* = 979, 52.20% girls). Adolescents’ frequency of checking social media, social media activity, and social media screen time were assessed using interviews and objective measures. Parents’ frequency of checking social media and problematic social media use were assessed using questionnaires. Adolescents reported on their closeness and conflicts with their parents. Attachment was measured by self-report and observation of parent-child interaction, while family functioning was assessed by both adolescent- and parent-report. Using random intercept cross-lagged panel modelling, no evidence was found for either between-family associations or within-family prospective links between adolescents’ and parents’ social media use and poorer quality of family relationships, family functioning, or attachment. These findings suggest that normative variation in social media use is unlikely to be a meaningful driver of changes in family relationship quality, and that concerns about everyday social media use harming family life may not be warranted.

## Introduction

A campaign called “The Pause Box” has been run by the telecommunication company Telia, encouraging families to put their phones in a box at agreed-upon times, such as during meals. The idea is simple: the less screen time and distraction, the higher the quality of family time. But do high levels of social media use actually result in worse family relationships? The results of a systematic review (Liu et al., 2024) do suggest so. However, as detailed below, existing research is mostly cross-sectional and thus not positioned to test such an assumption. To overcome the shortcomings of existing studies, prospective relations are explored here by separating between- from within-family effects, accounting for time-invariant confounding using six waves of data from two birth cohorts of children followed from age 10 to 20, relying on interviews and questionnaires completed by participants and their parents, in addition to observational data and screen logs. Because different types of media and usages may have differential effects, several aspects of parents’ and adolescents’ social media use beyond overall use are examined in the present study: frequency of checking, social media activity, and objectively measured smartphone social media screen time. These were related to core aspects of the quality of family and adolescent-parent relationships: family functioning, adolescent-parent conflict and closeness, and the extent to which adolescents are securely attached to their parents.

### Adolescents’ and Parents’ Social Media Use and Family Relationships

Although the field has increasingly moved beyond time- and frequency-based indicators of social media use toward more nuanced characterizations of *what* users do online and *how* they engage (Valkenburg et al., 2022; Yang et al., 2021), and the displacement framework originally proposed by Kraut et al. (1998) has received limited support for many individual psychosocial outcomes (Sanders et al., 2024; Vasconcellos et al., 2025), it remains particularly relevant when the outcome of interest is family relationship quality. The mechanisms thought to link social media to *individual* outcomes such as mental well-being depend heavily on the qualitative features of use, but the mechanism by which social media could affect *relationships* between co-present family members is fundamentally about how time and attention are allocated. On this view, what matters is not the content of a parent’s or adolescent’s feed but whether the time spent on it comes at the expense of shared family time and reciprocal engagement.

Within this framing, Social Displacement Theory posits that time spent on social media displaces time spent on face-to-face interactions, thereby potentially reducing opportunities for quality family time (Kraut et al., 1998). The theory is relevant here because social media use within the family may reduce opportunities for a range of growth-enhancing interactions. These interactions have been proposed and empirically supported across family and developmental theories, including serving as a secure base (Bowlby, 1988; Waters & Cummings, 2000), sensitive responding (Ainsworth et al., 1974), scaffolding (Wood et al., 1976), and modelling self-regulation (Morris et al., 2007). Despite differences in terminology and proposed mechanisms, these perspectives converge on the premise that sustained shared attention and reciprocal engagement are prerequisites for growth-enhancing interaction. Extensive phone use, including phubbing and inattentiveness during shared time (e.g., meals, face-to-face conversations), may prevent parents from attending to the needs of their children and children from engaging with their parents. Collectively, these dynamics suggest that social media use within the family may disrupt the interactional routines, emotional exchanges, and reciprocal engagement that underpin positive family relationships.

An alternative view, grounded in domestication theory (Silverstone & Hirsch, 1992; Berker et al., 2006) is that social media use becomes *domesticated*, i.e., fitted into the routines of family life, just like reading books or doing family chores. Even though direct interaction is limited when reading a book, tidying one’s room, or watching and responding to a friend’s story on Snapchat, family members compensate for the absence by resuming or starting new interaction when available. From this perspective, families are not passive recipients of social media’s effects but active regulators of how it fits into their everyday rhythms and routines. According to this view, within-family changes in social media use should not systematically predict within-family changes in the quality of family relationships.

Empirical evidence to date has been mixed and is not well positioned to determine which of the two perspectives outlined above is supported. Broadly, recent meta-analyses have found only small associations between time-use measures of screen use and youth outcomes (Sanders et al., 2024; Vasconcellos et al., 2025). Two recent systematic reviews (Liu et al., 2024; Vossen et al., 2024) have identified 32 and 42 studies, respectively, investigating the cross-sectional association between factors related to family climate or relationships and different aspects of adolescents’ social media use. These studies consistently reported that adolescent social media use was negatively correlated with factors such as family life, family support, family relationships, family communication, and family dynamics, a pattern consistent with the displacement perspective. Such correlational results have limited value for drawing any causal implications on the ill-effects of social media use, as these associations may be due to (1) social media use predicting quality of family relationships; and/or (2) quality of family relationships predicting social media use (e.g., conflicted adolescent-parent relationship predicts more social media use); and/or (3) confounding (i.e., third variables explaining why there seemingly is an association). Additionally, with a few exceptions summarized below, all studies included in these reviews (Liu et al., 2024; Vossen et al., 2024) were based on adolescent reporting exclusively, raising the possibility that observed correlations are inflated by common-rater bias. Among the three existing cross-sectional studies that included reports from both parents and adolescents (Geurts et al., 2022; Özaslan et al., 2022; Shutzman & Gershy, 2023), the links between social media use and quality of family relationships were weaker or more mixed.

Adding to this research are two longitudinal studies that employed Cross-Lagged Panel Models (CLPM) using two waves of data. One of these found no longitudinal relations between family hostility and closeness and social media use among adolescent girls, but boys’ social media use predicted decreased family closeness a year later (Simpson et al., 2023). The other examined university students’ problematic social media use and family functioning and found no evidence that these were prospectively related (Lu et al., 2025). A third longitudinal study also exists, reporting that adolescents who displayed high levels of social media use reported higher family conflict and lower family support six months later compared to those in the low social media use subgroup (Vannucci & Ohannessian, 2019). Importantly, though, baseline levels were not accounted for in this study, thus the reported estimate might be due to initial associations carried forward through stability of the measures and thus not reflect prospective relations between the variables. Taken together, single-informant cross-sectional findings support the displacement perspective, but multi-informant studies yield weak or mixed support for it. The longitudinal evidence is sparse and inconsistent. Previous studies have therefore not been able to determine whether the displacement perspective or the alternative view better accounts for the relations between social media use and family relationships.

Importantly, even though traditional longitudinal approaches such as Cross-Lagged Panel Models (CLPMs) can illuminate the direction of influence between variables, they conflate between-subject associations and within-subject prospective relations (Hamaker et al., 2015). As pointed out by several authors (Keijsers, 2016; Murayama & Gfrörer, 2025), findings in CLPM often do not translate into pure within-person prospective relations. To illustrate, families where adolescents and parents use more social media on average than other families might display worse family relationships (a negative between-family association at the group level). However, even if such between-family effect exists, within the family, a specific family’s *increased* social media might predict a subsequent improvement in the quality of family relationships (a positive within-family effect). Thus, it is essential to separate between- from within-person effects to come closer to the nature of the relation between social media use and family relationships, as is the aim of the present study.

## Current Study

Despite growing concerns about the impact of social media use on family life, it remains unclear whether increased social media use forecasts changes in family relationship quality. Drawing on the displacement framework, within-family increases in adolescents’ and parents’ social media use should predict subsequent decreases in indicators of family functioning, relationship quality, and attachment. Existing tests of this expectation, however, have relied predominantly on cross-sectional designs and single-informant data, and the few longitudinal studies available have not separated stable between-family differences from within-family prospective effects, risking inflated associations and misinterpretation of between-family differences as developmental processes. The present study therefore tests this prediction by using multi-informant data, examining whether within-family changes in multiple aspects of social media use (i.e., frequency of checking, activity, screen time, problematic use) predict within-family changes in family relationship quality (i.e., family functioning, adolescent-parent relationships, attachment) across ages 10 to 20.

## Methods

### Participants and Procedure

The Trondheim Early Secure Study (TESS; Wichstrøm et al., 2012) started when the participants were 4 years old. Parents of all children born in 2003 and 2004 (*N* = 3,456) in Trondheim, Norway, received mailed invitations, along with the Norwegian version of the Strengths and Difficulties Questionnaire (SDQ; Goodman et al., 2000). Parents were asked to complete the SDQ and bring it to their 4-year-olds’ health checkup at their local well-child clinic (3,358 attended). Among these, 176 families were excluded due to limited Norwegian proficiency, and 166 families were missed being asked to participate by the health nurse, leaving 3,016 eligible families, of whom 2,477 (82.1%) consented to participate. Children with emotional or behavioral difficulties were oversampled to strengthen statistical power, as the project’s initial focus was on mental health. To achieve oversampling—which was accounted for in the statistical analyses—the children were allocated to four strata based on their SDQ scores (cut-offs: 0–4, 5–8, 9–11, 12–40), with higher scores increasing the probability of selection (0.37, 0.48, 0.70, and 0.89 in the four strata, respectively). Subsequently, a final sample of 1,250 families was drawn. At the first assessment (T1; age 4 years), 1,007 families attended the university clinic with their child and have been assessed biennially since. Ethical approval was granted by the Regional Committee for Medical and Health Research Ethics (Steinsbekk & Wichstrøm, 2018).

Measures of social media use were included from age 10 years onwards, thus the present study covers ages 10, 12, 14, 16, 18, and 20 years, with data collection running from 2013 to 2024. Data collection from parents was discontinued after participants reached age 18. Hence, data at age 20 were obtained exclusively from the young adult participants. The analytical sample included 979 participants (52.20% girls) with at least one available data point on any study variable. Attrition analyses indicated that retention at age 12 was not predicted by sex or any of the study variables at age 10. Retention at age 14 and 16 was predicted by higher parent-reported family functioning two years earlier (OR = 0.33, *p* = .018 & OR = 0.37, *p* = .008). Retention at age 18 was predicted by more social media screen time (OR = 1.04, *p* = .030) and at age 20 was predicted by female sex (OR = 3.15, *p* = .008). Given that 50 *p*-values were calculated across the attrition analyses, the false discovery rate was adjusted for using the Benjamini–Hochberg procedure. None of the four initially significant predictors remained significant after adjustment, suggesting that attrition was not robustly associated with the study variables. Additionally, although an overall test of missingness using Little’s Missingness Completely at Random (MCAR) test showed that the attrition was not missing completely at random (*χ*² = 15791.47, *df* = 14547, *p* < .001), the normed test was 1.09, suggesting that deviations from MCAR were not substantial.

### Measures

#### Adolescents’ Social Media Activity 

Social media activity from ages 10 to 20 was assessed as the frequency of posting updates and photos, as well as liking and commenting on others’ posts (Steinsbekk et al., 2021). These behaviors were measured through semi-structured interviews conducted by trained personnel, using predefined questions (e.g., “How often do you like others’ updates?”), response categories, prompts (e.g., “If you think about yesterday…”), and follow-up questions to facilitate accurate recall. At ages 16, 18, and 20, photo posting was assessed using two items distinguishing between selfies and non-selfies; these were summed to ensure comparability with the single photo-posting item used at earlier time points. Based on participants’ responses, the total frequency of these activities in the preceding month was estimated. Response categories were expanded at older ages to capture expected increases in frequency (e.g., a 5-point scale at age 12 and a 10-point scale at age 16, ranging from 1 = seldom or never to 10 = more than 60 times a day). Responses were subsequently recoded to align across time points, with higher values indicating greater frequency of social media activity.

#### Adolescents’ Frequency of Checking Social Media

The frequency of checking social media was also captured by the above-noted interview, measured at ages 10, 12, and 14 years. Participants were asked to report the number of times they checked popular platforms—in the morning, during school hours, after school and before bedtime, and after going to bed—on a typical day (e.g., “How many times do you check e.g., Facebook/ Twitter/ Instagram/ Snapchat after school/before going to bed on a typical day?”). Because checking social media during school hours is unlikely to affect the quality of adolescent-parent relationship, this period was excluded from the total checking score applied in the present study, which was calculated by summing the number of checking on social media in the remaining time periods.

#### Adolescents’ Objectively Measured Social Media Screen Time 

At ages 16, 18, and 20 years, this was measured using screen time applications on participants’ iPhones. During the interview, when participants were asked about their social media use, the social media screen time of those who owned iPhones was recorded by research assistants. The number of hours spent on social media applications during the last 7 days served as an objective indicator of social media use duration. Among participants who owned smartphones, the proportion using iPhones ranged from 81.3% to 88.5% across the three assessment waves, with the remainder using Android devices, which did not provide comparable screen time data.

#### Parents’ Frequency of Checking Social Media 

When participants were aged 10, 12, 14, 16, and 18 years, parents completed a questionnaire about their own social media use. They were asked to report the number of times they checked social media on a usual day across four distinct time intervals: before 08:00 in the morning, between 08:00 and 16:00, from 16:00 until bedtime, and after going to bed. Because checking social media during typical work hours (08:00–16:00) is unlikely to affect the quality of adolescent-parent relationship, this time period was excluded from the social media checking sum score applied here. Frequency of checking social media for each parent was calculated by summing up each parent’s number of checking social media in the remaining time periods. When calculating parents’ combined score, the following conditions were applied: If both parents had valid (i.e., non-missing) values for their total number of checking social media, the mean of both was used as parents’ score; if only one parent had valid value, that was counted as parents’ total score; if both parents’ number of checking social media was missing, the parents’ total score was counted as missing.

#### Parents’ Problematic Social Media Use 

When participants were aged 10, 12, 14, 16, and 18 years, parents responded to the following items: “I think I spend too much time on social media.”, “I find it difficult to limit my use of social media.”, “I feel addicted to social media.”, which was scored along a 5-point Likert scale (1 = completely disagree, 2 = disagree, 3 = neither agree nor disagree, 4 = agree, and 5 = completely agree). For the final item, “If you were not allowed to use social media for an entire week, how difficult do you think it would be?”, a different 5-point scale was used (1 = not at all difficult, 2 = a little difficult, 3 = quite difficult, 4 = very difficult, and 5 = terrible). Each parent’s problematic social media use score was computed as the mean of their responses to the four items. Mothers’ and fathers’ scores were then averaged to yield a combined parental score. When only one parent had valid (i.e., non-missing) problematic social media use score, that parent’s score was used as the parent’s total score; cases with missing scores from both parents were coded as missing. The scale showed good internal consistency for both parents (*α* = 0.82–0.87).

#### Family Functioning 

Both adolescents (across age 14–20 assessments) and parents (across age 10–18 assessments) responded to the 12-item General Functioning subscale of the 60-item McMaster Family Assessment Device (FAD; Epstein et al., 1983). Both the long (FAD) and short (FAD-GF) formats are reliable tools for measuring family climate and functioning (Staccini et al., 2015; Byles et al., 1988). Sample items are “We are able to make decisions about how to solve problems.” and “In times of crisis we can turn to each other for support.” The response format is reverse-worded in a 4-point scale (1 = strongly agree, 2 = agree, 3 = disagree, 4 = strongly disagree), such that higher total score (mean of all items) indicates worse family functioning (*α* parent = 0.86–0.89, *α* adolescent = 0.88–0.92).

#### Adolescent-Parent Relationship Closeness and Conflict 

Across ages 10 to 20 years, adolescents reported on their closeness and conflicts with their mothers and fathers by responding to four closeness-related items and six conflict-related items (e.g., “How often do you and these people disagree and argue?”) from the Network of Relationships Inventory (NRI; Furman & Buhrmester, 1985). Due to low measurement invariance, items 3 and 7 of the original six closeness items were excluded, and only the remaining four items were used. Items were rated on a 5-point scale (1 = *Never or almost never* to 5 = *Very often or always*). Mean scores were computed for both scales separately for mothers and fathers and then averaged to yield a total adolescent-parent conflict and adolescent-parent closeness score. The scale showed good internal consistency for both parents (*α* for conflict with mother and father ranging from 0.89 to 0.94, and 0.88 to 0.96, respectively, *α* for closeness to mother and father ranging from 0.78 to 0.88, and 0.81 to 0.90, respectively).

#### Adolescent Attachment Security 

At ages 10, 12, 14, and 16, participants completed the Norwegian version of the 15-item Security Scale (Kerns et al., 2001). Each item presents two contrasting descriptions (e.g., “Some children worry that their mother/father might not be there when they need her/him … but … other children are sure that their mother/father will be there when they need her/him”). The mean score across all items was calculated (range 1–4), with higher scores indicating greater attachment security (*α* = 0.77–0.86).

#### Adolescent Attachment Representation 

At ages 10, 12, and 14 attachment representation—referring to children’s internal working models of the caregiver and the self that guide expectations and behavior in close relationships—was measured using the Middle Childhood Attachment Strategies Coding System (MCAS; Brumariu et al., 2018), an observational coding system capturing children’s attachment-related behavioral strategies during structured parent–child interactions. Dyads completed a series of videotaped, developmentally appropriate interaction tasks designed to activate the attachment system. Each interaction was rated on six independent 9-point scales capturing the extent to which each attachment strategy characterized the child’s behavior (1 = none or minimal evidence; 9 = marked and persistent evidence). Children’s attachment strategies were coded across six forms of attachment patterns: *secure*,* ambivalent*,* avoidant*,* disorganized-disoriented*,* caregiving/role-confused*, and *hostile/punitive*. Coding was dimensional, with each strategy scored separately based on behavioral and affective markers within the interaction context. Coders were trained and blind to other study data. Consistent with prior validation work, dimensional scores were used rather than categorical classifications.

### Data Analysis

For descriptive purposes, bivariate correlations between adolescent and parent social media use characteristics and family relationship characteristics were estimated. Since these add up to 106 possible correlations, the Benjamini–Hochberg procedure was used to control for the false discovery rate. To test the main research questions regarding within-family prospective predictions, 26 Random Intercept Cross-Lagged Panel Models (RI-CLPM; Hamaker et al., 2015) were estimated. Due to marked positive skewness and extreme values, all social media use variables, except parents’ problematic use, were log-transformed in the RI-CLPMs to improve estimation stability. One RI-CLPM for each of the adolescents’ and parents’ social media use characteristics and each of the family relationship characteristics were conducted. Each model consisted of the following components: A random intercept for each pair of the study variables (e.g., adolescents’ social media activity and family functioning) loading on every observed score at each time point with the factor loading set to 1. These intercepts capture the stable, trait-like differences between subjects by representing each person’s or family’s average level across measurement occasions (i.e., between-family variations). For each observed measure, a corresponding latent construct was generated, which was assigned a loading of 1, and the error variance in the observed variable was set to 0. This approach relocates the variance in the observed variable to its latent counterpart. These latent variables represent within-person changes (i.e., deviations from a person’s own long-term average) at each time point. Additionally, contemporaneous correlations were also allowed between the residuals of these latent changes. Finally, the latent changes on each occasion were regressed on the latent changes of all variables from the preceding time point, enabling a within-family and within-person analysis using families and individuals as their own control.

All analyses were carried out using Mplus Version 8.5 (Muthén & Muthén, 1998–2017). Probability weights were included to adjust for the intentional oversampling of children with emotional and behavioral problems and to achieve corrected population estimates. A robust Maximum Likelihood Estimator (MLR) and a Full Information Maximum Likelihood (FIML) procedure were employed, ensuring that all available data was used.

## Results

Descriptive statistics for the study variables are presented in Table 1. After adjusting for the false discovery rate, two out of 106 bivariate cross-sectional associations remained significant (Table 2). These were between adolescent-parent conflict and social media frequency of checking (*r* = .21) and activity (*r* = .21). [Insert Tables 1 and 2 around here.] Results of the 26 RI-CLPMs showed that at the within-family level, only four paths were significant: (1) increased adolescent social media frequency of checking at age 12 predicted more secure adolescent attachment representation at age 14 (*β* = 0.19, *p* = .003, See model 5 in Table 3); (2) increased adolescent social media frequency of checking at age 12 predicted decreased adolescent-parent closeness at age 14 (*β* = − 0.14, *p* = .045, See model 3 in Table 3); (3) increased adolescent social media use activity at age 12 predicted decreased adolescent-parent closeness at age 14 (*β* = − 0.13, *p* = .020, See model 9 in Table 3); and (4) increased parental social media frequency of checking when their adolescent was 14 years old predicted less adolescent attachment security at age 16 (*β* = − 0.13, *p* = .034, See model 5 in Table 4). However, none of these within-family prospective cross-lagged paths remained significant after adjusting for false discovery rate. As shown in Tables 3 and 4, most nonsignificant within-family cross-lagged effects were very small in magnitude, with standardized beta coefficients ranging from − 0.10 to 0.10 and showed no consistent directional pattern. This indicates that, beyond their statistical non-significance, the estimated effects were also of negligible magnitude. At the between-family level, only one correlation remained significant after adjusting for false discovery rate: adolescent social media use activity correlated with adolescent-parent closeness across ages 10 to 20 years (*r* = .35, *p* = .001, adjusted *p* = .026). Taken together, the results of all 26 RI-CLPMs (Tables 3 and 4) consistently showed no robust evidence for associations between social media use and family relationship quality at either the between-family or within-family level. [Insert Tables 3 and 4 after this sentence.]

**Table 1:** Sample Description and Mean and Standard Deviation for Scores on Adolescents’ and Parents’ Social Media Use (SMU) Characteristics and the Family Relationship Characteristics (*n* = 979)

Nominal Age	10	12	14	16	18	20
Mean of Age	10.51 (0.17)	12.49 (0.15)	14.35 (0.16)	16.98* (0.31)	18.60 (0.24)	20.56 (0.27)
*Adolescents’ Social Media Use*						
Frequency of Checking	1.24 (4.64)	6.72 (14.43)	21.72 (50.50)	—	—	—
Activity	27.73 (44.18)	59.61 (83.72)	78.96 (54.38)	584.81 (761.79)	504.85 (630.10)	486.53 (625.00)
Screen Time	—	—	—	22.08 (10.47)	25.04 (11.26)	25.94 (36.08)
*Parents’ Social Media Use*						
Frequency of Checking	3.24 (5.45)	6.50 (14.42)	7.60 (15.35)	7.95 (7.71)	7.53 (5.87)	—
Problematic Use	1.96 (0.66)	2.15 (0.70)	2.25 (0.69)	2.35 (0.71)	2.38 (0.75)	—
*Quality of Family Relationships*						
Family Functioning (Parent-reported)	1.59 (0.39)	1.59 (0.38)	1.62 (0.39)	1.69 (0.39)	1.67 (0.39)	—
Family Functioning (Adolescent-reported)	—	—	1.76 (0.45)	1.75 (0.45)	1.69 (0.50)	1.60 (0.49)
Adolescent-Parent Conflict	1.90 (0.59)	1.95 (0.65)	2.03 (0.67)	2.12 (0.70)	2.02 (0.71)	2.12 (0.85)
Adolescent-Parent Closeness	4.63 (0.46)	4.67 (0.47)	4.50 (0.54)	4.32 (0.63)	4.26 (0.70)	4.42 (0.68)
Attachment Security	3.45 (0.36)	3.41 (0.37)	3.27 (0.40)	3.24 (0.43)	—	—
Attachment Representation	4.88 (2.18)	5.04 (1.94)	5.22 (1.93)	—	—	—

Note. The values within parentheses are standard deviations. *Data collection for age 16 was delayed due to the COVID-19 restrictions. *Measures*: Adolescents’ frequency of checking social media = total daily checking frequency (excluding school hours). Adolescents’ social media activity = total monthly sum of posting, liking, and commenting. Adolescents’ social media screen time = hours spent on social media applications during the past 7 days. Parents’ frequency of checking social media = total daily checking frequency (excluding work hours). Parents’ problematic social media use = mean of four items rated on 5-point scales (1 = completely disagree to 5 = completely agree; and 1 = not at all difficult to 5 = terrible) averaged across both parents; higher values indicate more problematic use. Family functioning = mean of 12 items rated on a 4-point scale (1 = strongly agree to 4 = strongly disagree); higher values indicate worse family functioning. Adolescent-parent closeness and conflict = mean scores rated on 5-point scales (1 = never or almost never to 5 = very often or always); averaged across both parents. Adolescent attachment security = mean of 15 items (range 1–4); higher values indicate greater attachment security. Adolescent attachment representation = six dimensional 9-point scales (1 = none or minimal evidence to 9 = marked and persistent evidence) capturing adolescents’ attachment-related behavioral strategies during structured parent-child interactions

**Table 2:** Bivariate Correlations between Adolescents’ and Parents’ Social Media Use (SMU) Characteristics and the Family Relationship Characteristics (*n* = 979)

Variables	Family Functioning	Family Functioning	Adolescent-Parent Conflict	Adolescent-Parent Closeness	Attachment Security	Attachment Representation
Source of Information	Parent	Adolescent	Adolescent	Adolescent	Adolescent	Observation
	Age 10					
Adolescent SMU Checking	0.018	—	− 0.016	0.026	− 0.017	− 0.011
Adolescent SMU Activity	− 0.074	—	− 0.001	0.036	0.020	− 0.078*
Parent SMU Checking	0.078	—	− 0.030	0.042	0.032	0.008
Parent Problematic SMU	0.108*	—	0.036	0.055	0.006	− 0.019
	Age 12					
Adolescent SMU Checking	0.027	—	0.205***	− 0.185	− 0.158*	0.033
Adolescent SMU Activity	− 0.030	—	0.086**	− 0.026	− 0.074	− 0.017
Parent SMU Checking	− 0.056	—	− 0.057	0.049*	0.048	0.062
Parent Problematic SMU	0.091*	—	− 0.003	0.080*	− 0.004	− 0.008
	Age 14					
Adolescent SMU Frequency	− 0.028	0.165**	0.089	− 0.165*	− 0.170**	− 0.099
Adolescent SMU Activity	0.010	0.117*	0.210***	− 0.027	− 0.065	0.107*
Parent SMU Checking	− 0.006	0.042	0.028	− 0.025	− 0.063	0.008
Parent Problematic SMU	0.064	0.000	0.063	0.038	0.006	− 0.018
	Age 16					
Adolescent SMU Activity	− 0.075	− 0.026	− 0.006	0.009	− 0.039	—
Adolescent SMU Screen Time	0.038	0.092	0.071	− 0.063	− 0.066	—
Parent SMU Checking	− 0.018	− 0.016	− 0.027	− 0.028	− 0.009	—
Parent Problematic SMU	− 0.032	− 0.034	0.000	− 0.002	− 0.068	—
	Age 18					
Adolescent SMU Activity	0.003	0.033	0.002	0.004	—	—
Adolescent SMU Screen Time	0.045	0.080	0.116*	− 0.045	—	—
Parent SMU Checking	0.037	0.059	0.035	− 0.017	—	—
Parent Problematic SMU	0.028	− 0.010	0.042	0.083	—	—
	Age 20					
Adolescent SMU Activity	—	0.059	0.083	− 0.002	—	—
Adolescent SMU Screen Time	—	0.104	0.124	− 0.030	—	—

Note. Only correlations in **bold** remained significant after controlling for the false discovery rate (Benjamini–Hochberg procedure) at *q* < 0.05 to account for multiple comparisons

Asterisks indicate unadjusted *p* values. *p < .05. **p < .01. ***p < .001

**Table 3:** Details of 14 RI-CLPMs of the Relations between Adolescents’ Social Media Use (SMU) Characteristics and Quality of Family Relationships

#	Variables	Ages	Model Fit Indices	Between-Family Correlation	Within-Family Cross-lagged Paths
1	Adolescent SMU Checking & Family Functioning (Parent-reported)	10–14	*n* = 727, *χ*^*2*^(1) = 0.00, *p* = .995, RMSEA < 0.001, SRMR < 0.001, CFI = 1.00, TLI = 1.00	*r* = − .02 *p* = .817	*β* = − 0.07 to 0.05 all *p*s ≥ 0.204
2	Adolescent SMU Checking & Adolescent-Parent Conflict	10–14	*n* = 727, *χ*^*2*^(1) = 0.52, *p* = .471, RMSEA < 0.001, SRMR = 0.006, CFI = 1.00, TLI = 1.00	*r* = .26 *p* = .091	*β* = − 0.07 to 0.08 all *p*s ≥ 0.120
3	Adolescent SMU Checking & Adolescent-Parent Closeness	10–14	*n* = 727, *χ*^*2*^(1) = 0.38, *p* = .536, RMSEA < 0.001, SRMR = 0.006, CFI = 1.00, TLI = 1.00	*r* = − .05 *p* = .674	*β* = − 0.14* to 0.18 all *p*s ≥ 0.045
4	Adolescent SMU Checking & Adolescent Attachment Security	10–14	*n* = 727, *χ*^*2*^(1) = 0.39, *p* = .535, RMSEA < 0.001, SRMR = 0.007, CFI = 1.00, TLI = 1.00	*r* = .02 *p* = .925	*β* = − 0.09 to − 0.02 all *p*s ≥ 0.156
5	Adolescent SMU Checking & Adolescent Attachment Representation	10–14	*n* = 727, *χ*^*2*^(1) = 0.25, *p* = .618, RMSEA < 0.001, SRMR = 0.004, CFI = 1.00, TLI = 1.00	*r* = − .07 *p* = .529	*β* = 0.03 to 0.19** *p*s = 0.003 to 0.628
6	Adolescent SMU Activity & Family Functioning (Parent-reported)	10–18	*n* = 810, *χ*^*2*^(21) = 23.99, *p* = .294, RMSEA = 0.013, SRMR = 0.029, CFI = 0.997, TLI = 0.995	*r* = − .07 *p* = .417	*β* = 0.00 to 0.08 all *p*s ≥ 0.149
7	Adolescent SMU Activity & Family Functioning (Adolescent-reported)	14–20	*n* = 930, *χ*^*2*^(9) = 26.16, *p* = .002, RMSEA = 0.045, SRMR = 0.030, CFI = 0.979, TLI = 0.936	*r* = − .20 *p* = .089	*β* = − 0.03 to 0.09 all *p*s ≥ 0.183
8	Adolescent SMU Activity & Adolescent-Parent Conflict	10–20	*n* = 975, *χ*^*2*^(37) = 72.45, *p* < .001, RMSEA = 0.031, SRMR = 0.037, CFI = 0.977, TLI = 0.959	*r* = .11 *p* = .290	*β* = − 0.04 to 0.10 all *p*s ≥ 0.051
9	Adolescent SMU Activity & Adolescent-Parent Closeness	10–20	*n* = 978, *χ*^*2*^(37) = 70.11, *p* < .001, RMSEA = 0.030, SRMR = 0.041, CFI = 0.976, TLI = 0.957	***r*** = .35** *p* = .001	*β* = − 0.13* to 0.08 all *p*s ≥ 0.020
10	Adolescent SMU Activity & Adolescent Attachment Security	10–16	*n* = 811, *χ*^*2*^(9) = 8.56, *p* = .479, RMSEA < 0.001, SRMR = 0.025, CFI = 1.00, TLI = 1.00	*r* = .24 *p* = .198	*β* = − 0.05 to 0.06 all *p*s ≥ 0.259
11	Adolescent SMU Activity & Adolescent Attachment Representation	10–14	*n* = 723, *χ*^*2*^(1) = 1.26, *p* = .261, RMSEA = 0.019, SRMR = 0.009, CFI = 0.999, TLI = 0.988	*r* = − .08 *p* = .704	*β* = − 0.02 to 0.13 all *p*s ≥ 0.072
12	Adolescent SMU Screen Time & Family Functioning (Adolescent-reported)	16–20	*n* = 878, *χ*^*2*^(1) = 1.42, *p* = .234, RMSEA = 0.022, SRMR = 0.011, CFI = 0.999, TLI = 0.986	*r* = .43 *p* = .434	*β* = − 0.06 to − 0.02 all *p*s ≥ 0.303
13	Adolescent SMU Screen Time & Adolescent-Parent Conflict	16–20	*n* = 884, *χ*^*2*^(1) = 0.09, *p* = .759, RMSEA < 0.001, SRMR = 0.004, CFI = 1.00, TLI = 1.00	*r* = .53 *p* = .431	*β* = − 0.11 to − 0.06 all *p*s ≥ 0.245
14	Adolescent SMU Screen Time & Adolescent-Parent Closeness	16–20	*n* = 882, *χ*^*2*^(1) = 0.36, *p* = .547, RMSEA < 0.001, SRMR = 0.006, CFI = 1.00, TLI = 1.00	*r* = .08 *p* = .842	*β* = 0.01 to 0.07 all *p*s ≥ 0.455

Note. Only correlations in **bold** remained significant after controlling for the false discovery rate (Benjamini–Hochberg procedure) at *q* < 0.05 to account for multiple comparisons

Asterisks indicate unadjusted *p* values. *p < .05. **p < .01. ***p < .001

**Table 4:** Details of 12 RI-CLPMs of the Relations between Parents’ Social Media Use (SMU) Characteristics and Quality of Family Relationships

#	Variables	Ages	Model Fit Indices	Between-Family Correlation	Within-Family Cross-lagged Paths
1	Parent SMU Checking & Family Functioning (Parent-reported)	10–18	*n* = 723, *χ*^*2*^(1) = 19.78, *p* = .535, RMSEA < 0.001, SRMR = 0.026, CFI = 1.00, TLI = 1.00	*r* = − .06 *p* = .365	*β* = − 0.09 to 0.09 all *p*s ≥ 0.155
2	Parent SMU Checking & Family Functioning (Adolescent-reported)	14–18	*n* = 743, *χ*^*2*^(1) = 0.09, *p* = .767, RMSEA < 0.001, SRMR = 0.003, CFI = 1.00, TLI = 1.00	*r* = − .05 *p* = .723	*β* = 0.04 to 0.19 all *p*s ≥ 0.111
3	Parent SMU Checking & Adolescent-Parent Conflict	10–18	*n* = 812, *χ*^*2*^(21) = 40.29, *p* = .007, RMSEA = 0.034, SRMR = 0.029, CFI = 0.987, TLI = 0.972	*r* = − .07 *p* = .446	*β* = − 0.02 to 0.05 all *p*s ≥ 0.435
4	Parent SMU Checking & Adolescent-Parent Closeness	10–18	*n* = 812, *χ*^*2*^(21) = 26.52, *p* = .188, RMSEA = 0.018, SRMR = 0.039, CFI = 0.996, TLI = 0.991	*r* = − .03 *p* = .668	*β* = − 0.04 to 0.11 all *p*s ≥ 0.149
5	Parent SMU Checking & Adolescent Attachment Security	10–16	*n* = 803, *χ*^*2*^(9) = 15.26, *p* = .084, RMSEA = 0.029, SRMR = 0.028, CFI = 0.994, TLI = 0.981	*r* = − .09 *p* = .389	*β* = − 0.13* to 0.07 all *p*s ≥ 0.034
6	Parent SMU Checking & Adolescent Attachment Representation	10–14	*n* = 723, *χ*^*2*^(1) = 0.32, *p* = .573, RMSEA < 0.001, SRMR = 0.004, CFI = 1.00, TLI = 1.00	*r* = .09 *p* = .430	*β* = − 0.11 to − 0.01 all *p*s ≥ 0.300
7	Parent Problematic SMU & Family Functioning (Parent-reported)	10–18	*n* = 720, *χ*^*2*^(21) = 19.91, *p* = .527, RMSEA < 0.001, SRMR = 0.028, CFI = 1.00, TLI = 1.00	*r* = .02 *p* = .692	*β* = − 0.05 to 0.08 all *p*s ≥ 0.234
8	Parent Problematic SMU & Family Functioning (Adolescent-reported)	14–18	*n* = 741, *χ*^*2*^(1) = 6.06, *p* = .014, RMSEA = 0.083, SRMR = 0.015, CFI = 0.992, TLI = 0.887	*r* = .03 *p* = .847	*β* = − 0.11 to 0.09 all *p*s ≥ 0.103
9	Parent Problematic SMU & Adolescent-Parent Conflict	10–18	*n* = 812, *χ*^*2*^(21) = 28.09, *p* = .138, RMSEA = 0.020, SRMR = 0.028, CFI = 0.996, TLI = 0.992	*r* = − .13 *p* = .144	*β* = 0.04 to 0.11 all *p*s ≥ 0.071
10	Parent Problematic SMU & Adolescent-Parent Closeness	10–18	*n* = 812, *χ*^*2*^(21) = 22.90, *p* = .350, RMSEA = 0.011, SRMR = 0.037, CFI = 0.999, TLI = 0.998	*r* = .12 *p* = .030*	*β* = − 0.13 to 0.07 all *p*s ≥ 0.092
11	Parent Problematic SMU & Adolescent Attachment Security	10–16	*n* = 802, *χ*^*2*^(9) = 7.64, *p* = .571, RMSEA < 0.001, SRMR = 0.027, CFI = 1.00, TLI = 1.00	*r* = .00 *p* = .963	*β* = − 0.06 to 0.09 all *p*s ≥ 0.145
12	Parent Problematic SMU & Adolescent Attachment Representation	10–14	*n* = 723, *χ*^*2*^(1) = 0.17, *p* = .683, RMSEA < 0.001, SRMR = 0.003, CFI = 1.00, TLI = 1.00	*r* = − .08 *p* = .513	*β* = − 0.02 to 0.11 all *p*s ≥ 0.268

Note. Only correlations in **bold** remained significant after controlling for the false discovery rate (Benjamini–Hochberg procedure) at *q* < 0.05 to account for multiple comparisons

Asterisks indicate unadjusted *p* values. *p < .05. **p < .01. ***p < .001

## Discussion

Social media has become deeply embedded in everyday family life, raising concerns that it may negatively affect the quality of family relationships by disrupting attention and communication, and decreasing face-to-face interaction. Previous studies have not been properly positioned to test this because they have mostly relied on cross-sectional designs. Two longitudinal studies exist, but these blended between- and within-family effects, thus the observed links could not determine whether increased social media use within a particular family would predict poorer family relationships within the same family. To extend existing evidence, the present study therefore analyzed within-family effects, applied multiple raters, assessed various aspects of social media use and family relationships, relying on observational and objective measures in addition to self-reports, while leveraging a large birth cohort followed biennially from ages 10 to 20. No evidence was found to support the assumption that in families where adolescents and parents increase their social media use, the long-term quality of their family relationships declines. There was also no evidence that such relations existed at the between-family level. More specifically, with one exception, adolescents and parents who displayed higher levels of social media use—irrespective of which aspect was assessed—were not more likely to report poorer family functioning, relationship qualities or attachment than others. Together, the breadth of social media and family relationship variables and the developmental period covered (ages 10 to 20) suggest that there may be no meaningful link within families between social media use and family relationship quality, though this does not preclude the possibility that more disruptive patterns of use, such as persistent withdrawal from shared family activities, could undermine family bonds.

Systematic reviews of cross-sectional studies conclude that concurrent correlations exist (Liu et al., 2024; Vossen et al., 2024). In the present study though, only two out of 106 potential cross-sectional associations between diverse measures of social media use and family relationships were found. Notably, all significant correlations emerged in same-rater associations (e.g., adolescent-reported social media use and family functioning) and not across raters (e.g., adolescent-reported social media use and parent-reported family functioning). This raises the question of whether associations found in former cross-sectional studies were influenced by confounding through common-rater bias, as most studies have relied on adolescents reporting on all measures, risking emergence of inflated correlations. This is partially supported by the notion that among existing studies that did include reports from both parents and adolescents (Geurts et al., 2022; Özaslan et al., 2022; Shutzman & Gershy, 2023), links between social media use and quality of family relationships were weaker or mixed. Previous longitudinal evidence has relied on traditional cross-lagged panel models and two waves of data, reporting either no prospective link between problematic social media use and family functioning (Lu et al., 2025) or only gendered prospective links (e.g., a link emerging only among boys, where more social media use predicts lower family closeness; Simpson et al., 2023). The present study adds by showing that when stable between-family differences are separated from within-family changes, there is no evidence to support the assumption that family members’ social media use prospectively affects family relationships over time.

Importantly, the measures of social media use employed here are largely generic indicators capturing how much adolescents and parents engage with social media, rather than *how*, *when*, or in *what* digital and offline context this engagement occurs. Scholars have recently argued that such generic measures obscure meaningful variation in social media use and are weak predictors of individual psychosocial outcomes (Valkenburg et al., 2022; Yang et al., 2021). A recent meta-analysis in the broader screen use literature points to a similar conclusion, with the authors recommending that screen time guidelines emphasize not only exposure time but also content quality and the social context of use (Vasconcellos et al., 2025). That said, the present study extends beyond typical time-use research by using several different measures (frequency of checking, activity, screen time, and problematic use), and supplementing self-report with objective measurement. Given that all these measures point to the same null finding, the more plausible explanation is not that the measures were too generic but that time spent on social media itself does not drive change in family relationships.

Several considerations help to interpret these null findings. While the present findings provide no evidence that the amount of social media use affects family relationship quality, they do not address whether the offline contextual conditions surrounding use may matter—whether use disrupts ongoing family conversation, whether it occurs in shared spaces or in private (e.g., adolescents withdrawing to their rooms), and whether interactions are resumed or repaired afterwards. These dimensions are not captured by generic time-use measures, including those used in this study, but are captured by constructs such as technoference—interruptions of face-to-face interactions caused by technology use (McDaniel & Radesky, 2018). Future research employing such constructs would help test whether any effect lies in moment-to-moment interruption rather than in total time on platforms.

Notably, even the parents’ problematic social media use measure, which moves beyond time use to capture compulsive engagement, did not predict family relationship quality. An important distinction here is that the problematic use scale assesses subjective perceptions of compulsion (e.g., feeling “addicted” to social media) rather than observable behavior during family interactions. One potential explanation for such null finding is that parents with problematic use may still be able to restrict social media use to times that do not interfere with family life, or to resume and repair interactions when present. This suggests that compulsive use may not translate into relational consequences unless it specifically intrudes on shared family time. Beyond time and frequency, the content of online experiences themselves may also shape family interaction. For example, distressing experiences such as cyberbullying may prompt some youth to seek closeness with parents while leading others to withdraw further. These content-based effects, like the contextual effects discussed above, could not be detected by the time-use measures applied here and would warrant explicit testing in future research.

Several limitations should be considered when interpreting the results of this study. First, the specific use of social media within a family context was not measured. Theoretical accounts often assume that social media disrupts attention and shared time, yet whether use occurred in the presence of other family members or during shared activities was not assessed (though use during school and work hours was excluded). When adolescents and parents use social media during meal time or spare time in their rooms (i.e., *when* they use it), whether their use during such moments is compulsive (i.e., *how* they use it in family contexts), and whether the family has specific rules or agreements about social media use (i.e., *what* the rules are) are all important for understanding the relations between social media and family relationships, but were beyond the scope of the present measures. Second, effects may operate over shorter time intervals than the biennial design captured. Family interactions unfold on a daily or even momentary basis, and effects of social media use may be immediate but transient, fading before the next biennial assessment. That said, given that very few concurrent bivariate correlations were found, it is likely that even shorter assessment intervals would have yielded minimal prospective within-person links. More fine-grained approaches are therefore needed, including large-scale observational studies that directly assess real-time interaction patterns within families, such as technoference and reciprocal or asynchronous forms of phubbing, rather than relying solely on self-reported frequency measures. Such approaches should capture these contextual aspects of use, employ shorter assessment intervals, and include data from all family members (e.g., siblings) to more precisely examine how these interaction dynamics unfold.

## Conclusion

The concern that everyday social media use displaces meaningful family interaction and may erode relationship quality is widely voiced, yet longitudinal tests have been scarce. Existing studies have rarely distinguished general differences between families—where families that use more social media also tend to have lower relationship quality—from changes within families, where a given family’s own increases in social media use precede declines in their relationships. Only the latter speaks to whether social media use actually drives change in family relationships over time. The present study addressed this gap using six biennial waves of multi-informant data from ages 10 to 20 and random intercept cross-lagged panel modelling. Across multiple aspects of adolescents’ and parents’ social media use and multiple indicators of family relationship quality, no evidence was found that within-family increases in social media use predict subsequent declines in family relationship quality. Effects may still emerge under specific conditions—during shared family time, through real-time interaction patterns such as reciprocal or asynchronous phubbing, or across shorter time lags than examined here. Nevertheless, the present findings do not support the view that increased social media use deteriorates family relationship quality.
